# Molecular Aspects of the Isolated Limb Infusion Procedure

**DOI:** 10.3390/biomedicines9020163

**Published:** 2021-02-07

**Authors:** Jüri Teras, Michael J. Carr, Jonathan S. Zager, Hidde M. Kroon

**Affiliations:** 1 Department of Surgical Oncology, North Estonia Medical Centre Foundation, 13419 Tallinn, Estonia; jyrite@gmail.com; 2 Tallinn University of Technology, 12616 Tallinn, Estonia; 3 Department of Cutaneous Oncology, Moffitt Cancer Center and Research Institute, Tampa, FL 33612, USA; Michael.Carr@moffitt.org (M.J.C.); Jonathan.Zager@moffitt.org (J.S.Z.); 4 Department of Oncologic Sciences, University of South Florida Morsani College of Medicine, Tampa, FL 33612, USA; 5 Department of Surgery, Royal Adelaide Hospital, Adelaide, SA 5000, Australia; 6 Faculty of Health and Medical Sciences, School of Medicine, University of Adelaide, Adelaide, SA 5000, Australia

**Keywords:** isolated limb infusion, melanoma, sarcoma, locally advanced melanoma and sarcoma, in-transit metastases, molecular aspects, melphalan, immunotherapy

## Abstract

For decades, isolated limb infusion (ILI) and hyperthermic isolated limb perfusion (HILP) have been used to treat melanoma in-transit metastases and unresectable sarcoma confined to the limb utilizing the effect of loco-regional high-dose chemotherapy to the isolated limb. Both procedures are able to provide high response rates in patients with numerous or bulky lesions in whom other loco-regional treatments are becoming ineffective. In comparison to systemic therapies, on the other hand, ILI and HILP have the advantage of not being associated with systemic side-effects. Although in principle ILI and HILP are similar procedures, ILI is technically simpler to perform and differs from HILP in that it takes advantage of the hypoxic and acidotic environment that develops in the isolated limb, potentiating anti-tumour activity of the cytotoxic agents melphalan +/− actinomycin-D. Due to its simplicity, ILI can be used in both preclinical and clinical studies to test new cytotoxic regimens and combinations with the aim to overcome tumour resistance. In the future, administration of cytotoxic agents by ILI, in combination with systemic treatments such as BRAF/MEK/KIT inhibitors, immunotherapy (CTLA-4 blockade), and/or programmed death (PD-1) pathway inhibitors, has the potential to improve responses further by inducing increased tumour cell death while limiting the ability of the tumour to suppress the immune response.

## 1. Introduction

Despite major developments in the recent years in the treatment of metastatic melanoma after the introduction of effective immunotherapy with checkpoint inhibitors and targeted therapies, in-transit metastases (ITMs) remain challenging to treat. Patients with ITMs may often not obtain the desired response to these systemic therapies while they can suffer severe systemic toxicity. On the other hand, ITMs can be too numerous and/or bulky to be effectively treated by local procedures such as surgical excision or intra-lesional injection [1]. 

Patients with an unresectable primary or recurrent sarcoma of the limb also constitute a treatment challenge. In earlier times, these patients underwent amputation of the affected limb; however, this does not reduce the metastatic rate or improve survival while it reduces the patient’s quality of life significantly. Similarly, systemic therapy, consisting of antracycline-based chemotherapy of doxyrubicin in combination with ifosfamide, and/or external beam radiation are often unsatisfactory with low overall survival (OS) rates and high associated toxicity [2,3]. 

In case locally advanced melanomas or sarcomas are confined to a limb, these patients can often be effectively treated by high-dose loco-regional chemotherapy administered by either hyperthermic isolated limb perfusion (HILP) or isolated limb infusion (ILI), although these procedures are not uniformly recommended by current American, European and Australian guidelines [1,4,5,6,7,8,9,10,11]. Both HILP and ILI are well-established treatments in the neoadjuvant setting to improve resectability for sarcoma, as well as an adjuvant or palliative treatment when melanoma ITMs or local recurrences of sarcoma are present. Despite their similar indications and comparable response rates, however, the principles of HILP and ILI are different due to their molecular working mechanism. 

### 1.1. Principles of Isolated Limb Infusion 

**Figure 1:**
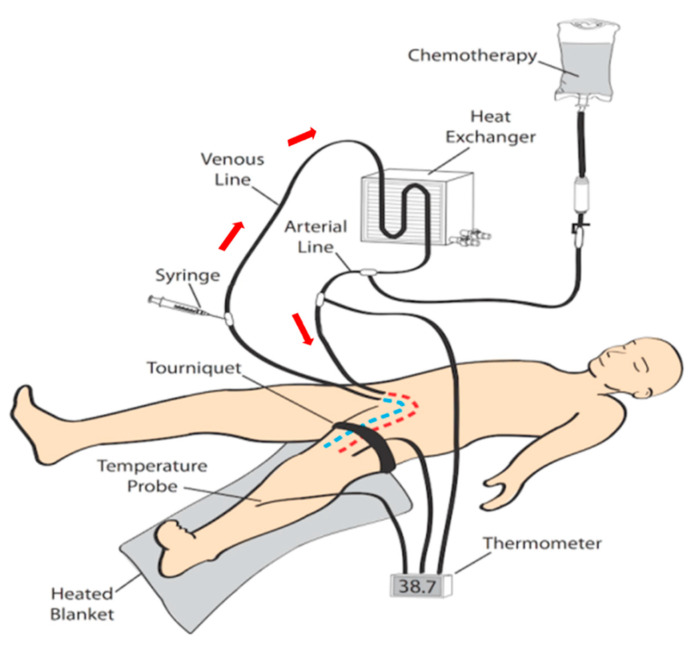
Schematic illustration of the circuit used for isolated infusion of a lower limb (red arrows indicate direction of flow of infusate).

**Figure 2:**
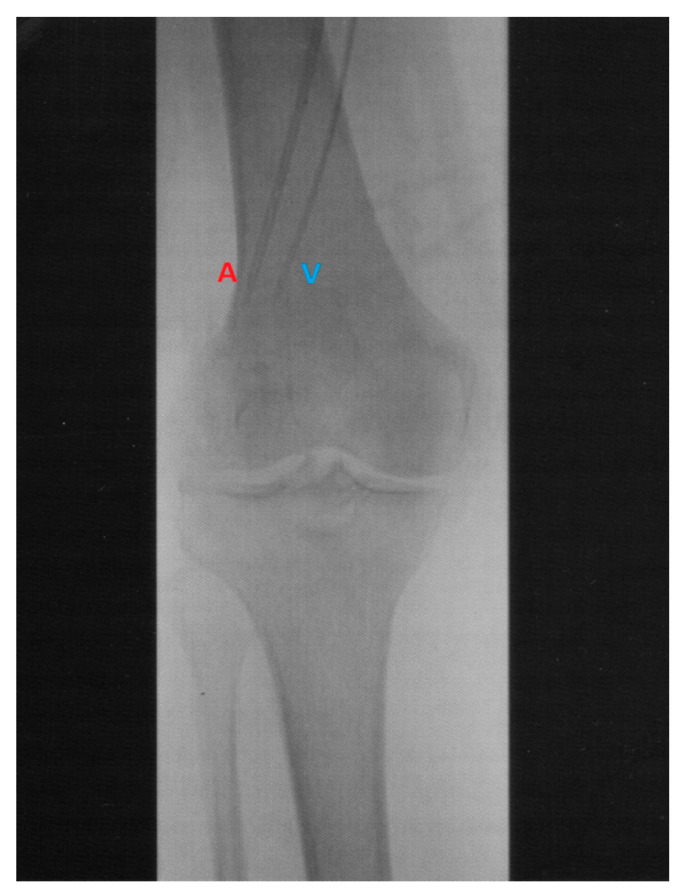
Angiogram of the arterial (A) and venous (V) catheters positioned in a lower limb with the tips reaching into the mid-popliteal vessels just proximal to the knee.

ILI was developed and implemented at the Melanoma Institute Australia (MIA, then called Sydney Melanoma Unit) in the 1990s as a simplified, minimally invasive alternative to HILP [12]. Today, ILI is used at multiple tertiary melanoma referral centres around the world [13,14]. Instead of open surgical cannulation as performed in HILP, percutaneously placed arterial and venous catheters are used to administer the cytotoxic agents into the affected limb (Figure 1; Figure 2) [15]. The catheters are placed using the Seldinger technique to the disease bearing limb which is isolated from the rest of the body by placement of the tourniquet at the level of groin or axillary fossa. The catheter tips are positioned just above the elbow or knee joint with tissue above the tips being perfused in a retrograde fashion via collateral vessels. During ILI, the infusate is not oxygenated, resulting in hypoxia and acidosis of the limb. Prior studies have shown that this hypoxic and acidotic environment enhances anti-tumour efficacy of the cytotoxic agents, normally melphalan in combination with actinomycin-D and may be advantageous to increase response rates [16,17]. Moreover, the low pressure and flow in the isolated limb (1000–1500 mL during the procedure, in HILP up to 10 times higher) allows for increased drug exposure of the tissues while avoiding systemic toxicity by minimal to no leakage of the cytotoxic infusate to the systemic circulation [18]. These measures, and the mild hyperthermia, improve the take up of the cytotoxic agents by the exposed tissues, augmenting tumour responses.

### 1.2. Patient Selection for ILI

Similar to HILP, patients with unresectable melanoma confined to the limb are considered eligible for the ILI procedure. Although ILI is mostly used for unresectable melanoma, it has also been used to treat patients with unresectable sarcoma, squamous cell carcinoma, Merkel cell carcinoma, refractory warts of the hands, refractory chromomycosis and localised refractory cutaneous T-cell lymphoma [16].

In general, the ILI procedure is well-tolerated. Even medically compromised, frail, and elderly patients can endure an ILI procedure well, making it feasible to treat many who would otherwise be considered unsuitable for treatments such as HILP or systemic therapy [19]. Particularly in elderly patients, ILI appears to be an attractive and safe procedure, since older patients experience less limb toxicity compared with younger patients (Wieberdink grade III/IV toxicity 36% vs. 51%; *p* = 0.009), while efficacy, systemic toxicity, complications, and long-term morbidity were similar in a multi-centre Australian study [20]. A recent study investigating ILI for melanoma ITMs in vulnerable octogenarian and nonagenarian patient showed that the procedure was safe and effective, with comparable responses and disease-control rates to younger patients [19]. Finally, in case of limb disease recurrences after an initial HILP or ILI, a repeat ILI can be considered with minimally increased limb toxicity or procedure-related morbidity [21]. 

### 1.3. Difference between ILI and ILP

ILI differs from HILP in that it is a minimally invasive procedure performed via percutaneously placed small-calibre catheters, whereas for HILP, a large invasive surgical procedure with open blood vessel cannulation using large-calibre catheters is required [5,15,22]. Due to the difference in catheter calibre between both procedures, blood circulation in the isolated extremity during ILI is at a much lower rate than HILP (50–100 mL/min in ILI vs. 150–1000 mL/min in HILP), and ILI drug exposure times are 30 min compared to 60 min in HILP. Another major difference is that the extremity during ILI is not oxygenated, leading to severe hypoxia and acidosis (Table 1). In contrast, during HILP, a pump oxygenator maintains oxygenation and a normal acid/base status of the extremity. The hypoxic conditions developing during ILI may in fact be advantageous by enhancing the cytotoxic effect since alkylating agents such as melphalan are more effective under these conditions. Furthermore, whereas blood transfusions are normally required during HILP to prime the extracorporeal circuit, this is not necessary in ILI. Moreover, if a patient has had previous groin or axillary surgery, for instance, a lymph node dissection, catheter insertion via the contralateral groin for ILI is usually straightforward, while both venous and arterial access for HILP can be technically difficult if not impossible, with both short- and long-term morbidity to vessel patency. Similarly, surgical access to the vessels for a repeat HILP procedure is often difficult due to scarring around the previous vascular access sites, while the percutaneous catheter insertion for a subsequent ILI procedure normally does not present problems. These and other differences between ILI and HILP are detailed in Table 2.

### 1.4. Challenges in Isolated Limb Infusion

Despite the simplicity of ILI, clinicians are faced with certain challenges associated with the procedure that will have to be overcome. Some of the technical challenges are discussed in this paragraph. For instance, placement of the venous catheter is sometimes difficult for both lower and upper limb ILI procedures because valves may be encountered near the root of the limb. However, it is usually possible to negotiate these valves by first passing a guide wire through them [15]. Furthermore, if a venous catheter of smaller calibre than 8FG is used, satisfactory venous return from the limb may be difficult to achieve. However, satisfactory venous return can be achieved using two 6FG catheters connected externally with a Y-connector when 8FG catheters are unavailable. It is also important that the patient is kept as warm as possible during the catheter insertion procedure and during transfer to the operating room or preoperative ward because low body and limb temperatures on arrival in the operating room make it more difficult to achieve adequate heating of the limb during the ILI procedure, limiting the chance of a favourable response. During ILI, melphalan tissue concentrations in the limb may vary between individual patients, making it challenging to estimate the correct dose for each patient. It is a matter of experience to overcome this particular challenge to know which patient, especially those with large (obese) limbs, may require an adjusted melphalan dose in order to achieve the best possible response without risking great limb toxicity [23,24]. In line with the experience in performing ILI, patient selection for the procedure is crucial to include all eligible patients, but also to be realistic about the likelihood for a patient to experience an advantageous response to the treatment in view of its associated limb toxicity. Assessing response can be difficult when the treated limb bears a large number of tumour nodules and when pigment remains after treatment. Additionally, response assessment can be complicated by the appearance of systemic disease, in which case patients often receive other forms of treatment that may have an effect on the magnitude or durability of response in the extremity. Overall, standardization of objective response criteria will aid future studies and will be necessary for valid comparisons between different studies, as now the WHO criteria for response and the RECIST criteria are used to report response to ILI by different groups, making comparisons difficult [1,25,26].

### 1.5. Toxicity Following Isolated Limb Infusion

To report limb toxicity, the Wieberdink toxicity scale, historically used for HILP, has shown to be also applicable after ILI [27]. Although after ILI reported limb toxicity is at the higher end of the spectrum of that reported for HILP, long-term morbidity is less frequently observed and less severe after ILI [28,29]. Considerable erythema and/or edema with blistering (grade III toxicity) is seen in around 19% of the patients. However, fasciotomies due to threatened or actual severe limb toxicity (grade IV toxicity) after ILI are only required in a small number of patients and from all reported series, only one patient has required an amputation due to toxicity (grade V limb toxicity). The majority of patients have no visible toxicity after ILI (grade I toxicity in 33%) or develop slight erythema and/or edema (grade II toxicity in 46%).

No relationship has been identified between increased limb toxicity and complete response (CR) to ILI, duration of response or survival, but a relationship was observed between limb toxicity and overall response, which likely shows the challenging balance between maximum efficacy while keeping toxicity low [29,30].

Various pharmacokinetic variables can predict limb toxicity after ILI. A high melphalan dosage, for instance, has been identified as a predictor of increased limb toxicity, and patients with longer ischemia times experience more severe limb toxicity [29,30]. Since uptake of melphalan is higher in muscle as opposed to fat, the skin and subcutaneous tissues of overweight patients are exposed to a relatively higher dose of melphalan [31]. In view of this, attempts have been made to lower the melphalan dose according to the patient’s ideal body weight (IBW), to reduce toxicity without jeopardising efficacy of the treatment [23,32].

After ILI, serious systemic side-effects, such as bone marrow depression or end-organ failure, are rarely seen and only mild postoperative nausea and vomiting is seen, which normally resolves quickly with conservative management [29,30]. The reason for the low number of patients with systemic toxicity after ILI can mainly be attributed to effective isolation placing a tourniquet around the base of the limb, while there is already low influx of chemotherapy from the isolated limb circuit to the systemic circulation due to the low flow and pressure in the isolated circuit of the to be treated limb.

### 1.6. Clinical Response Following Isolated Limb Infusion

In the majority of patients, cutaneous tumour deposits show signs of involution within 7–14 days following ILI. Sometimes, however, it can take several weeks before tumour deposits decrease appreciably in size.

The main goal of ILI is to achieve a CR, which is associated with increased OS [13]. Moreover, an overall response (complete and partial response combined) increases OS and improves the quality of life markedly [33]. After ILI, overall response rates of 64 to 73% have been reported, with a median OS of 38 to 101 months [1,13,28]. The reported overall response rates after HILP are somewhat higher, but it must be borne in mind that ILI is often performed in much older and fragile patients with more comorbidities [1,19].

### 1.7. Pharmacokinetics during Isolated Limb Infusion

**Figure 3:**
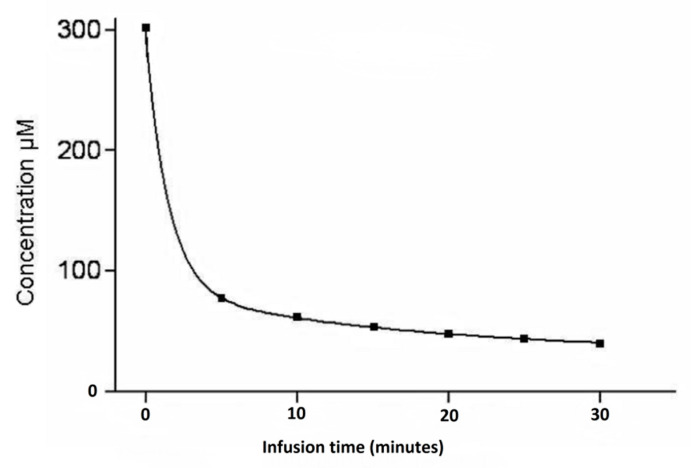
Typical melphalan concentration graph during isolated limb infusion.

Several drugs have been trialled for ILI, but the alkylating agent melphalan (L-phenylalanine mustard) +/− actinomycin-D is the most frequently used drug [1,28,34]. Melphalan has potent immunostimulatory properties, inducing a pro-inflammatory cytokine/chemokine environment acting as immunomodulator as well as directly inhibiting DNA replication of the tumour [35]. Melphalan achieves this by altering the tumour micro-environment through depletion of lymphocytic cells such as regulatory T cells and myeloid-derived suppressor cells and enhancing the release of pro-inflammatory cytokines and tumour antigen uptake by dendritic cells. In addition, a significant association between longer survival times, following locoregional melphalan chemotherapy in stage IIID melanoma patients with locoregional pelvic metastases, and a 14% cut-off value for O^6^-methylguanine-DNA methyltransferase (MGMT) promoter methylation has been reported [36]. During ILI, melphalan concentrations can be administered up to a 10-fold of the maximum tolerated systemic concentration, while systemic side-effects, such as end-organ toxicity and bone marrow depression are avoided by effective isolation achieved with placement of a tourniquet [18,37]. Normally, melphalan uptake by melanoma cells is a quick reaction (Figure 3) and occurs through active transport in a sodium and temperature dependent process, which plateaus after approximately 10 min into the procedure, while the elimination half-life of melphalan is 15 to 25 min [18,37,38]. Treatment of upper versus lower limb, location of tourniquet placement, circulation times, flow rates, circulated volumes, and limb temperatures are all variables that influence melphalan concentration in the infusate and its distribution in the isolated limb.

During ILI, Actinomycin-D is frequently used in combination with melphalan and acts as an antineoplastic antibiotic and interferes with transcription of DNA by RNA polymerase and modulates topoisomerase II activity. The combination of these two agents is believed to enhance response rates without compromising toxicity [1,39,40].

Drug dosage for ILI is calculated based on volume measurements of the tumour affected limb. Standard doses are 7.5 and 10 mg/L of melphalan for lower and upper limbs, respectively, and for actinomycin-D a standard dose of 75 µg/L for lower limb and 100 µg/L for upper limb is used [15]. Melphalan and actinomycin-D dosages may be corrected for IBW, which is commonly done in the US but not in Australian centres as several studies from this continent have addressed IBW correction, suggesting that melphalan dose correction does not decrease toxicity associated with ILI [23,32].

Microdialysis is a technique that potentially can be used during ILI to monitor drug concentrations in various tissues to investigate the relationship between the different concentrations in plasma, the interstitium, and tumour tissues [41,42]. By real-time monitoring of melphalan concentrations, microdialysis could help to optimise ILI conditions and improve tumour response. In a clinical HILP study conducted at the MIA, subcutaneous microdialysis catheters (CMA60/CMA70; CMA, Solna, Sweden) were inserted into normal and tumour tissues before commencement of the procedure. A microdialysis pump (CMA 106; CMA, Solna, Sweden) maintained constant infusion of fluids while melphalan concentrations in the samples were measured using high-performance liquid chromatography. Results of this study showed that peak plasma melphalan concentrations were higher than in subcutaneous and tumour tissues, allowing better understanding of the cytotoxic drug behaviour during HILP with the aim to improve postoperative limb morbidity and tumour response. Future studies are required to investigate the specific conditions during ILI. 

### 1.8. Isolated Limb Infusion as Platform to Test New Drugs and Drug Combinations

ILI is increasingly used as a platform to develop new drugs and drug delivery systems to increase intra-tumoural drug release [43,44]. Several clinical and preclinical studies have investigated combining agents to improve sensitivity of melanoma ITMs to chemotherapy. Melphalan with ADH-1, Sorafenib, Temozolomide, and Ipilimumab, for instance, have all shown a synergistic effect during ILI [45,46,47,48].

CTLA-4 blockade combined with melphalan ILI for melanoma ITMs, for instance, has shown a rapid response, suggesting that loco-regional chemotherapy can effectively be added to immunotherapy to potentiate the immune response [45]. The concept is that ILI can generate immune cell infiltration and increase the efficacy of CTLA-4 blockade. Although these results combining ILI and CTLA-4 blocking are promising, 38% of the patients experienced significant ipilimumab systemic side effects, a percentage similar to the 45% reported in large trials. ADH-1 is a cyclic pentapeptide that disrupts N-cadherin (regulating melanoma cellular proliferation, survival and angiogenesis) adhesion complexes. During ILI, combining ADH-1 with melphalan, improves tumour response by increasing drug delivery to melanoma cells, particularly in tumours that have become resistant to melphalan [46]. DNA-methylating agent Temozolomide (TMZ) is converted to 5-[3-methyl-triazen-1-yl]-imidazole-4-carboxamide (MTIC) during ILI, which overcomes chemo-resistance to melphalan by inhibiting O^6^ alkylguanine alkyl transferase. Patients with progressive disease after melphalan ILI have shown a response after TMZ ILI treatment [47]. Further studies on the use and efficacy of TMZ will be required to optimise regional responses. The multikinase inhibitor Sorafenib, a first generation BRAF inhibitor, antagonises RAF serine/threonine kinases and receptor tyrosine kinases, reducing tumour proliferation and tumour cell survival [48]. Concurrent administration of systemic Sorafenib during melphalan ILI reduces the tumour’s ability to suppress the immune response while it activates antigen-specific immune cells, inducing tumour death. Moreover, Sorafenib enhances sensitivity to chemotherapy by changing signalling in the mitogen-activated protein kinase and mitochondrial apoptotic pathways independent of the patient’s BRAF mutational status.

These studies show the relation between melphalan and tumour immunogenicity and suggest that in the future there may be a place for concurrent or sequential administration of systemic targeted therapies or immunotherapies with ILI. Finally, precision oncotherapy is under investigation, and drug regimens may be selected by chemosensitivity tests on purified circulating tumour cells (CTCs) obtained from liquid biopsies in individual patients [49].

### 1.9. Hyperthermia during Isolated Limb Infusion

**Figure 4:**
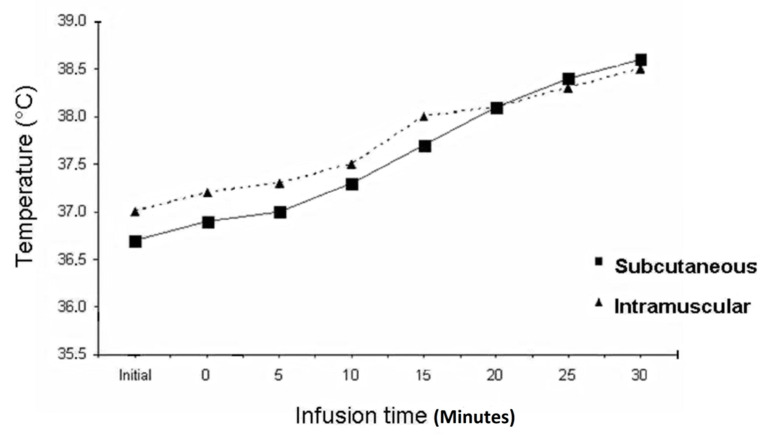
Typical temperature graph during isolated limb infusion.

ILI is performed under mild to moderate hyperthermic conditions (38–40 °C; Figure 4; Table 1). This is achieved by heating the infusate, by external heating of the affected limb with a radiant heater placed over it, and by applying a warm air blanket forming a cocoon around it [15]. Mild hyperthermia makes the tumour’s vascularity permeable for chemotherapy and elevates ATP release from erythrocytes, contributing to deep tissue perfusion by manipulating the supply of oxygen, nutrients, and regulatory substances [50]. Moreover, mild hyperthermia diminishes DNA repair, induces DNA interstrand cross-links, and inhibits cycline-dependent kinase activity by increasing tyrosine phosphorylation of the protein, potentiating the cytotoxic effect of melphalan [17,18].

True hyperthermia (>41 °C) causes further vascular damage and cell apoptosis, increasing chemo- and radiosensitivity of tumours; however, it also causes rapid degradation of melphalan resulting in reduced effective concentrations and higher toxicity rates [18,24,51,52].

Preclinical ILI studies using nanoparticle-based drug delivery systems in combination with true hyperthermia have shown good responses [44]. Whether exposing thermosensitive liposomes to true hyperthermia is also clinically safe and effective will have to be explored in future studies.

### 1.10. Hypoxia and Acidosis during Isolated Limb Infusion

**Figure 5:**
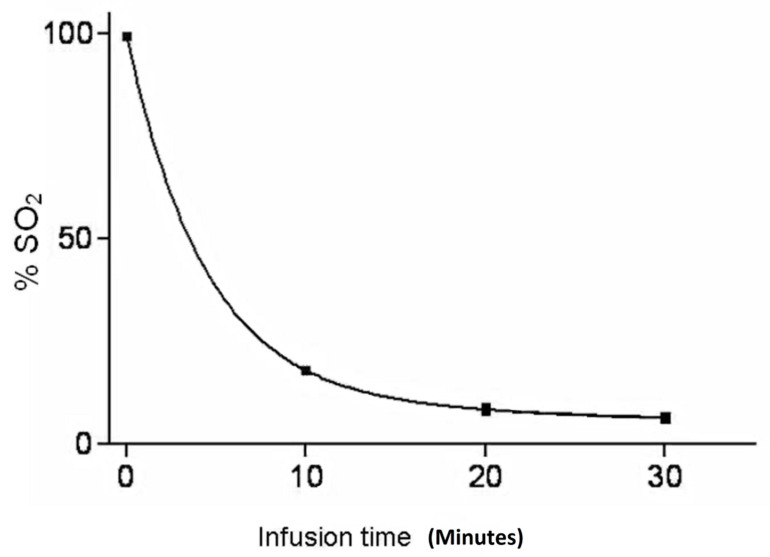
Typical saturation graph of the perfusate during isolated limb infusion.

During ILI, the limb becomes increasingly acidotic, hypoxic, and ischemic due to isolation with a tourniquet without using oxygenation (Figure 5; Table 1) [16]. Hypoxia reduces the cytotoxic immune response, enhancing the efficacy of melphalan by a factor of 1.5, whereas the combination of hypoxia and acidosis increases this effect by a factor of 3 [53,54]. This added effect of acidosis is caused by intensified cellular melphalan uptake, reducing the spontaneous hydrolysis rate and increased levels of nitric oxide in the tumour’s micro-environment. This has been confirmed in clinical studies showing improved response rates in patients with longer tourniquet times and whose blood taken from the isolated limb had increased CO_2_ levels [20,29,55].

### 1.11. Special ILI Regimens and Indications

Since its introduction, several special ILI regimens have been developed. These include a planned double procedure, a repeat ILI procedure for disease recurrence, ILI for palliation in patients with AJCC stage IV disease, and ILI as induction therapy [21,30,56,57].

A repeat procedure can benefit patients with recurrent or progressive disease after a first ILI if they had a favourable response to the initial ILI [21]. On the other hand, in patients who did not respond to the initial ILI, a repeat procedure is unlikely to provide benefit. In those cases, alternative loco-regional treatments like injection of intra-lesional agents, topical treatment with laser ablation or cryotherapy or inclusion in a trial to test systemic therapies can be considered. ILI can also be considered in a palliative setting to avoid limb amputation to achieve limb salvage and increase quality of remaining life in patients with both symptomatic limb disease as well as distant melanoma metastases [30]. Another mechanism by which ILI can help achieve limb preservation is by using it as induction therapy. Using this approach, ILI can convert unresectable disease to lesions amendable for simple local treatments by excision, laser ablation, electrodessication or injection with Rose Bengal (PV-10) [57]. A study investigating this approach has shown that after a PR to ILI, excision of residual melanoma lesions resulted in limb recurrence-free interval and OS rates similarly to those achieved following a CR after ILI alone [58].

### 1.12. Difference between Isolated Limb Infusion for Melanoma and Sarcoma

In patients with an extremity soft tissue sarcoma, ILI can achieve a limb salvage rate of up to 80% [59,60]. When performing sarcoma ILI, the same principles apply as for melanoma ILI; however, some adjust the procedure to achieve peripheral vasoconstriction, increasing blood-flow to the deep tissues where the sarcomas are mostly located. This in contrast to the peripheral vasodilatation pursued in melanoma ILI. The alteration of blood flow can be achieved not using papaverine and removing the preoperative external heating of the limb [16]. Interestingly, prognostic factors for sarcoma ILI associated with improved responses were low preoperative skin temperatures and a greater increase of temperatures during the procedure.

### 1.13. Novel Isolated Limb Infusion Regimens

In view of its minimally invasive character, the easy visual assessment of tumour response and simple access for biopsies of ITMs, ILI provides an ideal platform to explore novel therapeutic agents and therapy approaches [43]. Melphalan ILI in combination with intra-lesional agents, such as T-VEC or PV-10 for instance, can enhance response due to the so-called bystander effect, caused by the tumour antigen release and T cell activation by the immunological reaction caused by the intra-lesional agent [57]. Immune checkpoint inhibitors have improved the prognosis for patients with locally advanced melanoma, making ILI plus checkpoint inhibition a potential novel therapeutic strategy for patients with limb melanoma [45,61]. Moreover, doxorubicin ILI combined with external beam radiotherapy has the potential to increase local disease control [62].

### 1.14. Future of Isolated Limb Infusion

Understanding tumour immunology will help future selection of optimal drug strategy during ILI. Levels of immune activating cytokines are lower in patients with melanoma ITMs compared to healthy individuals, supporting a potential role for immune-targeted therapies and immune monitoring [63]. These considerations support the opinion that loco-regional chemotherapy still has a place in the treatment of advanced stage melanoma patients [64]. In the future, more is to be expected of immunotherapy combined with local chemotherapy via ILI to increase responses in locoregional melanoma.

For extremity sarcoma, the results of the currently recruiting trial combining ILI with Pembrolizumab will improve the knowledge of combinational treatments (NCT04332874). In addition, there is no published literature available on the effects of the use of external beam radiotherapy in sarcoma ILI, which in HILP has shown to reduce the risk for local recurrences especially when resection margins are close or microscopically positive [65].

## 2. Conclusions

ILI provides a simple and minimally invasive treatment to provide satisfactory and durable responses for treating melanoma ITMs and unresectable soft-tissue sarcoma. Measuring cytotoxic drug concentrations and biochemical processes in the tumour’s micro-environment helps gain understanding of the mechanisms of toxicity, tumour response, and chemoresistance and allows further improvement of the procedure and drug combinations used.

## Figures and Tables

**Table 1:** Median intra-operative values at 30 min upon completion of ILI in 185 patients at Melanoma Institute Australia [13].

Ph	7.11
Base excess (mmol/L)	−10.8
PO_2_ (mmHg)	8.4
SO_2_ (%)	6.9
PCO_2_ (mmHg)	54.3
Peak subcutaneous temperature (°C)	38.1
Peak intramuscular temperature (°C)	38.2
Drug exposure time (mins)	30
Tourniquet time (mins)	55

**Table 2:** Differences between hyperthermic isolated limb perfusion and isolated limb infusion.

Hyperthermic Isolated Limb Perfusion	Isolated Limb Infusion
Technically complex	Technically simple
Open surgical exposure of vessels for catheter insertion	Percutaneous vascular catheter insertion in radiology department
4 to 6 h duration	Approximately 1 h
Perfusionist and ancillary staff required	No perfusionist required and fewer total staff
Complex and expensive equipment needed	Equipment requirements modest
Magnitude of procedure excludes patients	Well tolerated by medically compromised, frail and elderly patients
Not possible in occlusive vascular disease	Can be performed selectively in occlusive vascular disease
Technically challenging to perform a repeat procedure	Not difficult to perform a repeat procedure
Systemic metastases normally a contraindication	Systemic metastases not a contraindication
Higher perfusion pressures predispose to systemic leakage	Low pressure system, effective vascular isolation with tourniquet
Limb tissues oxygenated, with normal blood gases maintained	Progressive hypoxia and acidosis
Hyperthermia (>41 °C can be achieved)	Usually not possible to raise limb temperature above 40 °C
General anesthesia (GA) required	Possible with regional anaesthesia, GA preferred.

## Data Availability

Not applicable.
